# Effects of Combustible Cigarettes and Heated Tobacco Products on Systemic Inflammatory Response in Patients with Chronic Inflammatory Diseases

**DOI:** 10.3390/diseases12070144

**Published:** 2024-07-05

**Authors:** Nikolina Kastratovic, Natasa Zdravkovic, Ivan Cekerevac, Vanesa Sekerus, Carl Randall Harrell, Violeta Mladenovic, Aleksandar Djukic, Ana Volarevic, Marija Brankovic, Tijana Gmizic, Marija Zdravkovic, Jelica Bjekic-Macut, Nebojsa Zdravkovic, Valentin Djonov, Vladislav Volarevic

**Affiliations:** 1 Center for Research on Harmful Effects of Biological and Chemical Hazards, Faculty of Medical Sciences, University of Kragujevac, 69 Svetozar Markovic Street, 34000 Kragujevac, Serbia; n_kastratovic@outlook.com (N.K.); natasasilvester@gmail.com (N.Z.); icekerevac@gmail.com (I.C.); ana.volarevic@fmn.kg.ac.rs (A.V.); nzdravkovic@gmail.com (N.Z.); 2 Department of Genetics, Faculty of Medical Sciences, University of Kragujevac, 69 Svetozar Markovic Street, 34000 Kragujevac, Serbia; 3 Department of Internal Medicine, Faculty of Medical Sciences, University of Kragujevac, 69 Svetozar Markovic Street, 34000 Kragujevac, Serbia; vikicam2004@gmail.com (V.M.); adjukic@fmn.kg.ac.rs (A.D.); 4 Center for Endocrinology, Diabetes and Metabolic Diseases, University Clinical Center Kragujevac, 30 Zmaj Jovina Street, 34000 Kragujevac, Serbia; 5 Pulmonology Clinic, University Clinical Center Kragujevac, 30 Zmaj Jovina Street, 34000 Kragujevac, Serbia; 6 Institute for Pulmonary Diseases of Vojvodina, 4 Institutski Put, 21204 Novi Sad, Serbia; vanesa.sekerus@mf.uns.ac.rs; 7 Department of Biochemistry, Faculty of Medicine, University of Novi Sad, 3 Hajduk Veljkova Street, 21000 Novi Sad, Serbia; 8 Regenerative Processing Plant, LLC, 34176 US Highway 19 N, Palm Harbor, FL 34684, USA; dr.harrell@regenerativeplant.org; 9 Department of Psychology, Faculty of Medical Sciences, University of Kragujevac, 69 Svetozar Markovic Street, 34000 Kragujevac, Serbia; 10 Department of Gastroenterology, University Medical Center “Bežanijska Kosa”, Dr Zoza Matea bb, 11080 Belgrade, Serbia; brankovic.marija@bkosa.edu.rs (M.B.); tijana93@hotmail.com (T.G.); 11 Faculty of Medicine, University of Belgrade, Dr Subotica 8, 11000 Belgrade, Serbia; sekcija.kardioloska@gmail.com; 12 Department of Cardiology, University Medical Center “Bežanijska Kosa”, Dr Zoza Matea bb, 11080 Belgrade, Serbia; 13 Department of Endocrinology, University Medical Center “Bežanijska Kosa”, Dr Zoza Matea bb, 11080 Belgrade, Serbia; jbjekic@yahoo.com; 14 Department of Statistics, Faculty of Medical Sciences, University of Kragujevac, 69 Svetozar Markovic Street, 34000 Kragujevac, Serbia; 15 Institute of Anatomy, University of Bern, Baltzerstrasse 2, 3012 Bern, Switzerland; valentin.djonov@unibe.ch; 16 Department of Microbiology and Immunology, Faculty of Medical Sciences, University of Kragujevac, 69 Svetozar Markovic Street, 34000 Kragujevac, Serbia; 17 Faculty of Pharmacy Novi Sad, Trg Mladenaca 5, 21000 Novi Sad, Serbia

**Keywords:** combustible cigarettes, heated tobacco products, systemic inflammation, cytokines, chronic inflammatory diseases

## Abstract

Smoke derived from combustible cigarettes (CCs) contains numerous harmful chemicals that can impair the viability, proliferation, and activation of immune cells, affecting the progression of chronic inflammatory diseases. In order to avoid the detrimental effects of cigarette smoking, many CC users have replaced CCs with heated tobacco products (HTPs). Due to different methods of tobacco processing, CC-sourced smoke and HTP-derived aerosols contain different chemical constituents. With the exception of nicotine, HTP-sourced aerosols contain significantly lower amounts of harmful constituents than CC-derived smoke. Since HTP-dependent effects on immune-cell-driven inflammation are still unknown, herein we used flow cytometry analysis, intracellular staining, and an enzyme-linked immunosorbent assay to determine the impact of CCs and HTPs on systemic inflammatory response in patients suffering from ulcerative colitis (UC), diabetes mellitus (DM), and chronic obstructive pulmonary disease (COPD). Both CCs and HTPs significantly modulated cytokine production in circulating immune cells, affecting the systemic inflammatory response in COPD, DM, and UC patients. Compared to CCs, HTPs had weaker capacity to induce the synthesis of inflammatory cytokines (IFN-γ, IL-1β, IL-5, IL-6, IL-12, IL-23, IL-17, TNF-α), but more efficiently induced the production of immunosuppressive IL-10 and IL-35. Additionally, HTPs significantly enhanced the synthesis of pro-fibrotic TGF-β. The continuous use of CCs and HTPs aggravated immune-cell-driven systemic inflammation in COPD and DM patients, but not in UC patients, suggesting that the immunomodulatory effects of CC-derived smoke and HTP-sourced aerosols are disease-specific, and need to be determined for specific immune-cell-driven inflammatory diseases.

## 1. Introduction

The systemic inflammatory response has a profound impact on the progression and exacerbation of chronic inflammatory diseases [1]. Prolonged systemic inflammation results in tissue damage and fibrosis in various organs [1,2]. The continuous activation of immune cells and the massive release of inflammatory mediators results in the destruction of parenchymal and endothelial cells (ECs) throughout the body, exacerbating the pathology of chronic inflammatory conditions [3]. Sustained inflammatory signaling is usually associated with impaired tissue repair and an excessive deposition of collagen in the vital organs, resulting in their dysfunction [2,3]. Additionally, systemic inflammation can trigger or exacerbate autoimmune response [4]. A detrimental systemic inflammatory response can disrupt the balance between pro-inflammatory and anti-inflammatory immune cells in injured tissues, leading to immune dysregulation and impaired immune tolerance [4]. A dysregulated immune response leads to the massive release of self-antigens from injured parenchymal cells, followed by the activation of autoreactive lymphocytes, resulting in the development and progression of autoimmune diseases [1,4].

Accordingly, the identification of factors that induce systemic inflammatory response and the mitigation of their detrimental effects could prevent exacerbations of inflammation-related diseases [1]. Prolonged exposure to combustible cigarette (CC)-derived smoke is considered an important risk factor for the induction of systemic inflammatory response and for the development of organ-specific immune-cell-driven diseases [5,6,7,8,9,10]. Smoke from CCs contains many harmful substances that can negatively impact the ability of immune cells to survive, proliferate, produce inflammatory cytokines, and migrate in injured tissues [11]. Neutrophils exposed to CC-sourced smoke had altered migratory characteristics and were not able to optimally produce reactive oxygen species (ROS) [12]. Similarly, CC-derived smoke favored an alternative activation of macrophages and suppressed their phagocytic ability against pathogenic bacteria [9]. A decrease in the number of M1 inflammatory macrophages was observed in healthy CC users compared to healthy non-smokers. The transition towards the M2-polarization phenotype was found to be more prominent in CC users who suffer from chronic obstructive pulmonary disease (COPD) [13]. High, but not low, levels of CC-derived smoke suppressed allergic airway inflammation in mice [14]. This suppression was achieved by inhibiting the function of T cells, reducing eosinophilia and decreasing the levels of IL-4 and IL-5 in the bronchoalveolar lavage [14]. Additionally, the smoke exposure led to a loss of ovalbumin antigen-specific proliferation and cytokine production in T cells [14]. Chronic exposure to CC-derived smoke may lead to a progression from M1 macrophage-dependent oxidative burst response to M2 macrophage-driven sensitization [14]. However, this M2 macrophage-driven immune response may be suppressed due to the loss of T-cell function and the suppression of IL-4 and IL-5 production [14]. Long-term exposure to cigarette smoking altered the production of perforins and granzymes in cytotoxic T lymphocytes (CTLs) and natural killer (NK) cells, affecting their anti-viral properties [10]. Additionally, increased expressions of E and P selectins were observed on ECs that were exposed to CC-derived smoke, facilitating the migration of circulating leukocytes in injured and inflamed tissues [7]. Accordingly, cigarette smoking is considered the main driver of inflammatory and malignant diseases of the lungs (COPD, pulmonary fibrosis, and lung cancer) and represents one of the main risk factors for the aggravation of immune-cell-driven organ-specific and systemic diseases [15]. The frequent use of CCs increases the risk of developing heart disease, stroke, peripheral arterial disease, atherosclerosis, diabetes, osteoporosis, and rheumatoid arthritis [15].

In order to avoid the detrimental effects of cigarette smoking, many CC users have replaced CCs with heated tobacco products (HTPs), which heat tobacco instead of burning it [16,17]. HTPs consist of a stick, capsule, or cartridge of tobacco that is inserted into a device that heats the tobacco to release nicotine and flavor without producing smoke [16]. The tobacco in HTPs is heated to a specific temperature, usually between 250 and 350 °C [16,17]. This temperature is enough to release nicotine and other chemicals without generating harmful combustion byproducts [16,17]. Due to the different methods of tobacco processing, CC-sourced smoke and HTP-derived aerosols contain different chemical constituents [18]. With the exception of nicotine, HTP-derived aerosols contain different concentrations of harmful and potentially harmful constituents than CC-derived smoke [17,18]. CC-derived tar, carbon monoxide (CO), benzene, 1, 3 butadiene, acrylonitrile, and ethylene oxide are present in lower amounts in HTP-sourced aerosols, but concentrations of several bioactive substances are higher in HTP-derived emissions compared to CC-sourced smoke, indicating that cessation from CCs and HTPs is the most effective strategy in the prevention of inflammatory and malignant diseases [18]. Compared to CC-derived smoke, HTP-sourced aerosols had different impacts on the phenotypes and functions of immune cells that infiltrated the lungs of COPD mice and COPD patients [5,19]. HTP-exposed lung dendritic cells (DCs) had impaired capacity for the optimal activation of inflammatory Th1, Th2, and Th17 lymphocytes compared to DCs that were continuously exposed to CCs [5]. Similarly, a reduced activation of the NLRP3 inflammasome was observed in the neutrophils and monocytes of HTP-exposed COPD mice and COPD patients [5,19]. Despite the fact that DC:T cell crosstalk and the NLRP3-dependent activation of circulating leukocytes are crucially responsible for the development of systemic immune response in patients suffering from COPD, ulcerative colitis (UC), and diabetes mellitus (DM), the long-term effects of HTPs on immune-cell-driven systemic inflammation are still unknown [16]. Accordingly, in this study, we analyzed the effects of CCs and HTPs on cytokine production in the circulating immune cells of patients suffering from these chronic inflammatory diseases, and we compared concentrations of inflammatory and immunosuppressive cytokines in their serum samples, paving the way to a better understanding of CC- and HTP-dependent effects on the immune-cell-driven systemic inflammatory response. 

## 2. Material and Methods

### 2.1. Study Population

In total, 74 patients with ulcerative colitis (UC), 78 patients with chronic obstructive pulmonary disease (COPD), and 33 patients with diabetes mellitus (DM) were recruited in this study. The diagnosis of UC was confirmed by endoscopic, histological, and clinical scores. Endoscopic disease activity was evaluated with the Mayo endoscopic subscore. The clinical score was determined using the Truelove and Witts clinical activity index. The histological score was determined using Geboes grade. The inclusion criterion for COPD patients was the diagnosis of airflow limitation that was not fully reversible. The diagnosis was confirmed by spirometry. The presence of a post-bronchodilator forced expiratory volume in 1 s (FEV_1_) < 80% of the predicted value, in combination with an FEV_1_/forced vital capacity (FVC) ratio < 70% confirmed the presence of airflow limitation that was not fully reversible. The inclusion criterion for DM was fasting glycaemia equal or greater than 126 mg/dL.

The patients were recruited from the Centers of Gastroenterology, Endocrinology and Pulmonology, University Clinical Center of Kragujevac, Centers of Gastroenterology and Endocrinology, University Clinical Center “Bezanijska Kosa” Belgrade, and from the Institute for Pulmonary Diseases of Vojvodina, Sremska Kamenica. UC, COPD, and DM patients who used CCs and HTPs were recruited to experimental groups (UC^CC^, COPD^CC^, DM^CC^, UC^HTP^, COPD^HTP^, and DM^HTP^), while UC, COPD and DM non-smokers were recruited to the UC^AIR^, COPD^AIR^, DM^AIR^ groups. Healthy volunteers who used CCs or HTPs or were non-smokers were stratified in three control groups (CCs, HTPs, and AIR). Participants from the experimental and control groups used 10–15 cigarettes or HTP heatsticks per day for at least one year. CC and HTP dual users were not included in the study. Information about the duration and frequency of CC/HTP usage was based on a self-reported questionnaire signed by each study participant. All participants from the experimental and control groups were matched according to age, gender, duration, and frequency of CC and HTP usage. An exclusion criterion for all groups was if participants had previously been diagnosed with any other disease instead of the one that was being investigated. Precisely, this included pregnancy, organ-specific autoimmune diseases (Addison’s disease, autoimmune hepatitis, coeliac disease, Grave’s disease, Guillain–Barre syndrome, Hashimoto’s thyroiditis, Crohn’s disease, multiple sclerosis, myasthenia gravis, pernicious anemia, primary biliary cholangitis, sclerosing cholangitis, etc.), systemic autoimmune diseases (systemic lupus erythematosus, polymyositis, dermatomyositis, rheumatoid arthritis, systemic sclerosis, Sjögren’s syndrome, etc.), psoriasis, fatty liver disease, liver cirrhosis, hepatitis A-E, and malignant and other life-treating diseases (heart failure, arrythmias, renal insufficiency, respiratory insufficiency, fulminant liver injury, etc.). 

All experiments were approved by the local Ethics Committees of University Clinical Center of Kragujevac (No. 01/22-341), University Clinical Center “Bezanijska Kosa” (No. 1711/1), and the Institute for Pulmonary Diseases of Vojvodina, Sremska Kamenica (No. 21-IV/3). The Declaration of Helsinki and *Principles of Good Clinical Practice* were always followed. Each patient provided informed consent before having their blood analyzed. 

### 2.2. Isolation of Immune Cells 

For the isolation of immune cells from the study participants, blood samples were obtained in heparinized collection tubes. Leukocytes were separated from erythrocytes and dead cells by Ficoll Paque (Amersham Biosciences, Otelfingen, Switzerland) gradient centrifugation, as previously described [20].

### 2.3. Intracellular Staining and Flow Cytometry Analysis of Immune Cells 

Isolated immune cells (1 × 10^6^ cells per sample) were stained for the intracellular content of TNF-α, IFN-γ, TGF-β, IL-1β, IL-4, IL-5, IL-6, IL-10, IL-12, IL-13, IL-17, IL-22, IL-23 and IL-35 by using the fixation/permeabilization kit and appropriate anti-human monoclonal antibodies conjugated with fluorescein isothiocyanate (FITC), phycoerythrin (PE), peridinin chlorophyll protein (PerCP), or allophycocyanin (APC) (BD Biosciences, San Jose, CA, USA) [21]. Flow cytometric analysis was conducted on a BD Biosciences FACSCalibur. The acquired data were analyzed using the Flowing software analysis program (version 2.5.1; Turku Bioscience Centre, Turku, Finland).

### 2.4. Measurement of Cytokines in Serum Samples 

The concentrations of inflammatory and immunosuppressive cytokines (TNF-α, IFN-γ, TGF-β, IL-1β, IL-4, IL-5, IL-6, IL-10, IL-12, IL-13, IL-17, IL-22, IL-23, and IL-35) were determined in the serum samples previously obtained from the study participants. For this purpose, the blood samples were centrifuged for 15 min, and then the serum was removed and kept at −80 °C. Commercial enzyme-linked immunosorbent assay (ELISA) kits (R&D Systems, Minneapolis, MN, USA) were used in accordance with the manufacturer’s instructions.

### 2.5. Statistics

Statistical analyses were performed using SPSS 21.0 for Windows software (SPSS, Inc., Chicago, IL, USA). The distribution of the data was checked by the Shapiro–Wilk or Kolmogorov–Smirnov test. Differences between mean values within groups were determined using one-way ANOVA. In cases when ANOVA identified a significant difference, individual differences were examined using two tailed Student’s *t* tests with a Tukey correction for multiple comparisons. The normality of the data distribution was investigated by Kolmogorov–Smirnov or Shapiro–Wilk tests according to the number of patients. After testing for normality, correlations were tested with the appropriate correlation coefficient: Pearson or Spearman’s. A multivariate linear regression model was used to discover deeper relations between clinical factors and measured levels of cytokines in the serum of the patients. The level of significance was *p* < 0.05. Data are expressed as the mean ± SEM for each group. All reported *p* values were 2-sided, and *p* < 0.05 was considered statistically significant.

## 3. Results

### 3.1. Continuous Use of Either CCs or HTPs Modulates Cytokine Production in Immune Cells of COPD Patients 

**Figure 1:**
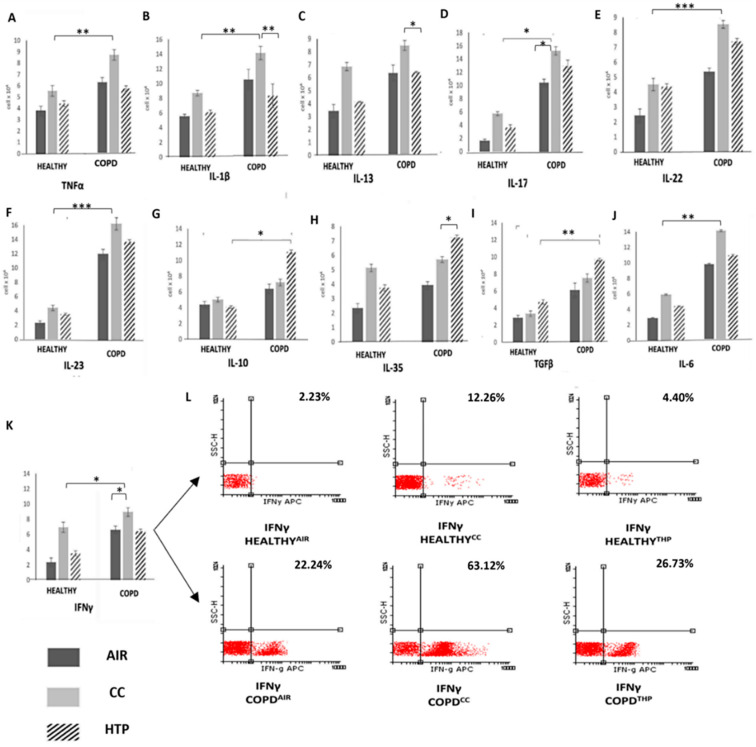
Continuous use of either CCs or HTPs modulates cytokine production in immune cells of COPD patients. Total numbers of TNF-α- (**A**), IL-1β- (**B**), IL-13- (**C**), IL-17- (**D**), IL-22- (**E**), IL-23- (**F**), IL-10- (**G**), IL-35- (**H**), TGF-β- (**I**), IL-6- (**J**), and IFN-γ-producing immune cells (**K**) in the blood of healthy volunteers and COPD^CC^, COPD^HTP^, and COPD^AIR^ patients, including representative dot plots (**L**), demonstrating altered capacity for cytokine production in leukocytes of COPD patients who used CCs or HTPs. Values are presented as mean ± SEM; * *p* < 0.05, ** *p* < 0.01, *** *p* < 0.001.

**Figure 2:**
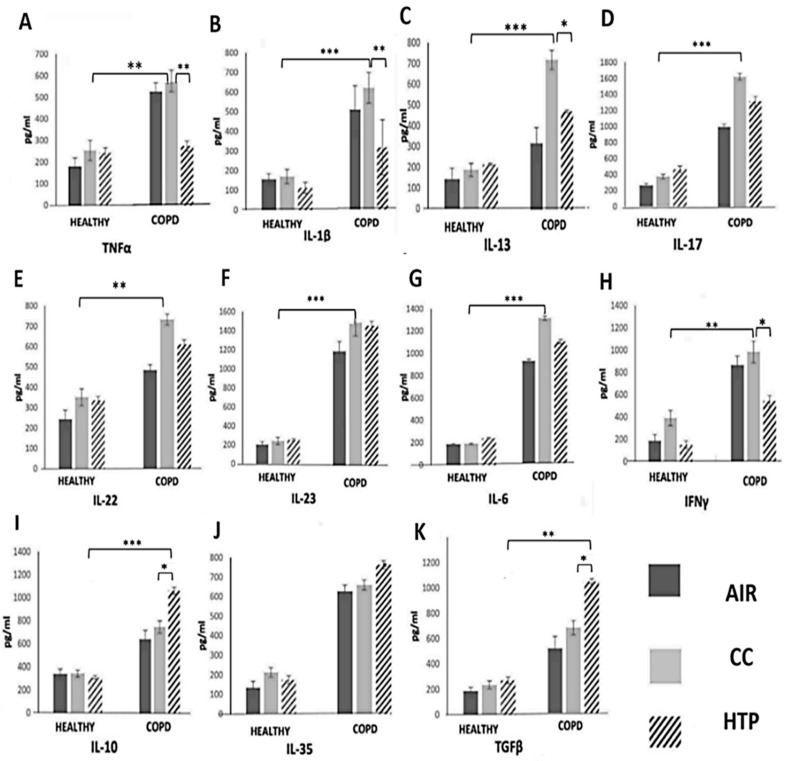
CC- and HTP-dependent effects on serum levels of inflammatory, pro-fibrotic, and immunosuppressive cytokines in healthy volunteers and COPD patients. Concentrations of TNF-α (**A**), IL-1β (**B**), IL-13 (**C**), IL-17 (**D**), IL-22 (**E**), IL-23 (**F**), IL-6 (**G**), IFNγ- (**H**), IL-10 (**I**), IL-35 (**J**), and TGF-β (**K**) in serum samples of healthy volunteers and COPD^CC^, COPD^HTP^, and COPD^AIR^ patients show that both CCs and HTPs altered production of inflammatory, immunosuppressive, and pro-fibrotic cytokines in COPD patients. Values are presented as mean ± SEM; * *p* < 0.05, ** *p* < 0.01, *** *p* < 0.001.

Lung-infiltrated immune cells, through the production of TNF-α, IL-1β, IL-6, IL-13, IL-17, IL-22, and IL-23, induce chronic lung inflammation, mucous hypersecretion, and ciliary dysfunction, which lead to airflow obstruction and hyperinflation that are clinically diagnosed by decreased FEV1 (COPD^AIR^ (74.57 ± 5.34) vs. COPD^CCs^ (61.97 ± 7.12) and COPD^HTPs^ (66.75 ± 4.94)) [22]. In line with these findings, we observed significantly higher numbers of inflammatory cells, as well as TNF-α-, IL-1β-, IL-13-, IL-17-, IL-22-, and IL-23-producing immune cells (Figure 1), and elevated levels of TNF-α, IL-1β, IL-13, IL-17, IL-22, and IL-23 in the serum samples of COPD patients compared to healthy individuals (Figure 2). Also, there was a positive correlation between the serum concentrations of these inflammatory cytokines and FEV1 (correlation coefficients were in the range of 0.133–0.403; *p* < 0.05), and a negative correlation between FEV1 and the serum levels of immunosuppressive IL-35 (correlation coefficients were in the range of values 0.064–0.642 with *p* < 0.05). Additionally, multivariate linear regression (adjusted R square = 28.2%, F = 7.887; *p* < 0.001) showed that the continuous use of CCs had a significant effect on FEV1 values (FEV1 values were higher than 0.728 in CC group, compared to AIR group).

By affecting cytokine production in immune cells, both HTPs and CCs altered the systemic inflammatory response in COPD patients. An increased number of immunosuppressive, IL-10-, TGF-β-, and IL-35-producing immune cells (Figure 1) and an elevated serum level of TGF-β were observed in COPD^HTP^ compared to COPD^AIR^ patients. The continuous use of HTPs also increased the production of Th17 cell-derived inflammatory cytokines (IL-17, IL-22) in COPD patients (Figure 1). Similarly, the long-term use of CCs increased the production of inflammatory cytokines, which importantly contributed to the development of airflow obstruction in COPD^CC^ patients, affecting COPD development and progression. A significantly higher number of IL-17- and IFN-γ-producing immune cells (Figure 1) and increased serum levels of TNF-α, IL-1β, IL-17, IL-23, and IL-13 (Figure 2) were observed in COPD^CC^ compared to COPD^AIR^ patients. 

Importantly, CCs and HTPs differed in their immunomodulatory properties. Higher numbers of TNF-α-, IL-1β-, IL-6-, IL-17-, and IFN-γ- producing leukocytes (Figure 1) and increased serum concentrations of TNF-α, IL-1β, IL-6, IFN-γ, IL-17, and IL-22 (Figure 2) were observed in COPD^CC^ compared to COPD^HTP^ patients. CCs had weaker capacity than HTPs to induce the synthesis of immunosuppressive cytokines (IL-10, IL-35) and pro-fibrotic TGF-β in COPD patients. Higher numbers of IL-10- and TGF-β-producing immune cells and remarkably higher serum levels of IL-10, IL-35, and TGF-β were observed in COPD^HTP^ compared to COPD^CC^ patients. 

### 3.2. Compared to CCs, HTPs Had Weaker Capacity to Induce Production of Th1- and Th17-Related Cytokines in Immune Cells of DM Patients

TNF-α and IL-1β are inflammatory cytokines that attract circulating leukocytes in injured pancreatic islets, importantly contributing to the aggravation of DM [23]. We observed a positive correlation between the serum concentrations of TNF-α and IL-1β and parameters of DM progression (glycemia and glycohemoglobin (HbA1c)) in DM patients who used CCs or HTPs (correlation coefficients were in the range of 0.066–0.849; *p* < 0.05). Additionally, serum levels of immunosuppressive IL-35, which attenuate the systemic inflammatory response, negatively correlated with HbA1c in DM^CC^ and DM^HTP^ patients (correlation coefficients were in the range of 0.383–0.775 with *p* < 0.05). Furthermore, multivariate linear regression (adjusted R square = 62.5%, F = 42.594; *p* < 0.001) showed that the continuous use of CCs had a significant effect on glycemia (values higher than 0.153 in CC group compared to AIR group) and HbA1c (values higher than 0.09 in CC group compared to AIR group).

**Figure 3:**
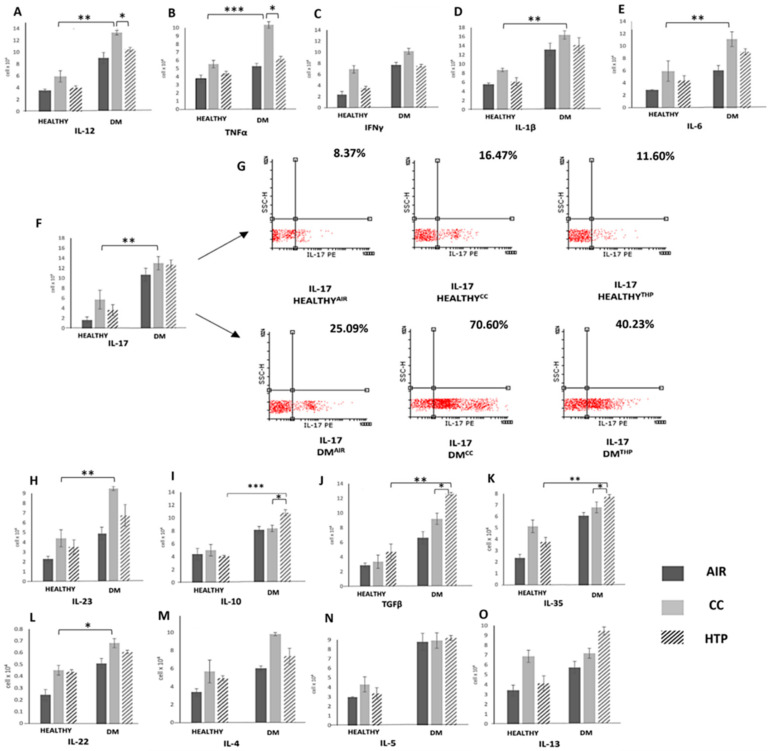
CC- and HTP-based modulation of cytokine production in circulating immune cells of DM patients. Total numbers of immune cells producing inflammatory, Th1-related (IL-12 (**A**), TNF-α (**B**), IFN-γ (**C**)) and Th17-related cytokines (IL-1β (**D**), IL-6 (**E**), IL-17 (**F**), IL-23 (**H**)) were lower and total numbers of immunosuppressive, IL-10- (**I**), TGF-β- (**J**), and IL-35-producing (**K**) immune cells were higher in the blood of DM^HTP^ than in the blood of DM^CC^ patients, indicating that, compared to CCs, HTPs had weaker capacity to induce the production of Th1- and Th17-related cytokines in the blood of DM patients. There was no statistical difference in the amount of IL-22 (**L**), IL-4 (**M**), IL-5 (**N**), or IL-13 (**O**) between DM^HTP^ and DM^CC^ patients. Representative dot plots show IL-17-producing cells (**G**). Values are presented as mean ± SEM; * *p* < 0.05, ** *p* < 0.01, *** *p* < 0.001.

**Figure 4:**
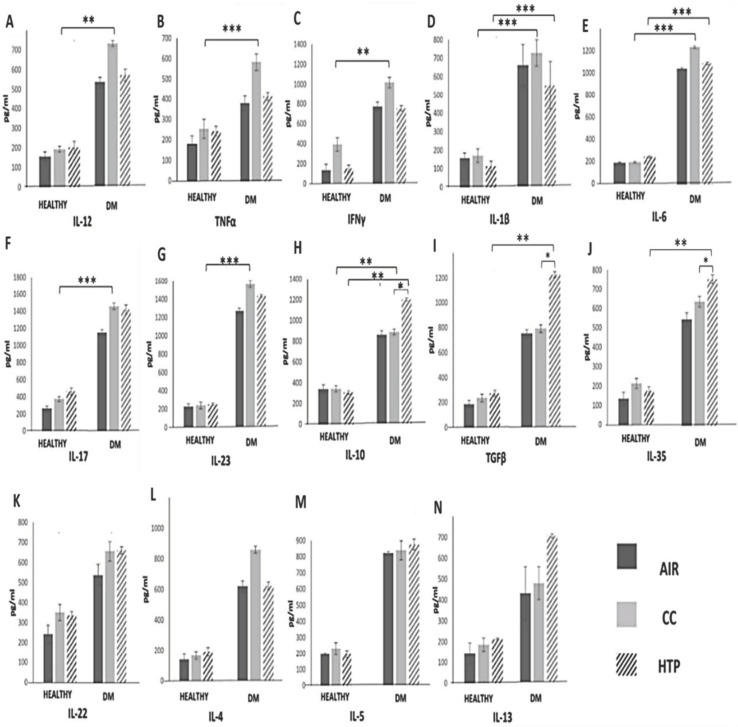
Effects of CCs and HTPs on serum levels of inflammatory and immunosuppressive cytokines in healthy individuals and DM patients. Concentrations of Th1-related cytokines (IL-12 (**A**), TNF-α (**B**), IFN-γ (**C**)) and Th17-related cytokines (IL-1β (**D**), IL-6 (**E**), IL-17 (**F**), IL-23 (**G**)) in serum samples of healthy volunteers and DM^CC^, DM^HTP^, and DM^AIR^ patients show that, in DM patients, CCs more efficiently enhanced the production of inflammatory, Th1-, and Th17-related cytokines compared to HTPs. CCs had weaker capacity than HTPs to induce the production of pro-fibrotic TGF-β (**I**) and immunosuppressive IL-10 (**H**) and IL-35 (**J**). There was no statistical difference in the concentrations of IL-22 (**K**) and Th2-related cytokines (IL-4 (**L**), IL-5 (**M**), IL-13 (**N**)) between serum samples of DM^HTP^ and DM^CC^ patients. Values are presented as mean ± SEM; * *p* < 0.05, ** *p* < 0.01, *** *p* < 0.001.

Th1 and Th17 cells have an important pathogenic role in the destruction of insulin-producing pancreatic beta cells [24]. Accordingly, increased serum levels of Th1- and Th17-related inflammatory cytokines crucially contribute to the generation of systemic inflammatory response in DM patients and the development of DM-associated complications [24]. We noticed an increased number of immune cells that produced Th1- (IL-12, TNF-α, IFN-γ) and Th17-related cytokines (IL-1β, IL-6, IL-17, IL-23) (Figure 3) in the blood of DM^CC^ and DM^HTP^ compared to DM^AIR^ patients, and we observed increased concentrations of these inflammatory cytokines in the serum samples of DM^CC^ and DM^HTP^ patients (Figure 4). Compared to CCs, HTPs had a weaker capacity to induce the production of inflammatory, Th1-, and Th17-related cytokines in DM patients. Lower numbers of TNF-α-, IL-12-, IFN-γ-, IL-1β-, IL-6-, and IL-17-producing immune cells (Figure 3) and lower serum levels of TNF-α, IL-12, IFN-γ, IL-1β, IL-6, IL-17 (Figure 4) were observed in DM^HTP^ than in DM^CC^ patients. 

Compared to CCs, HTPs more efficiently induced the expansion of immunosuppressive, IL-10- and TGF-β-producing leukocytes, which was manifested with increased concentrations of IL-10 and TGF-β in the serum samples of DM^HTP^ compared to DM^CC^ and DM^AIR^ patients. Also, HTPs increased the serum concentration of immunosuppressive IL-35, which was higher in DM^HTP^ than in DM^CC^ patients. Both CCs and HTPs increased the synthesis of IL-22 and did not significantly alter the production of Th2-related cytokines (IL-4, IL-5, IL-13) in DM patients.

### 3.3. Compared to CCs, HTPs More Efficiently Induce Production of Immunosuppressive Cytokines in Immune Cells of UC Patients

**Figure 5:**
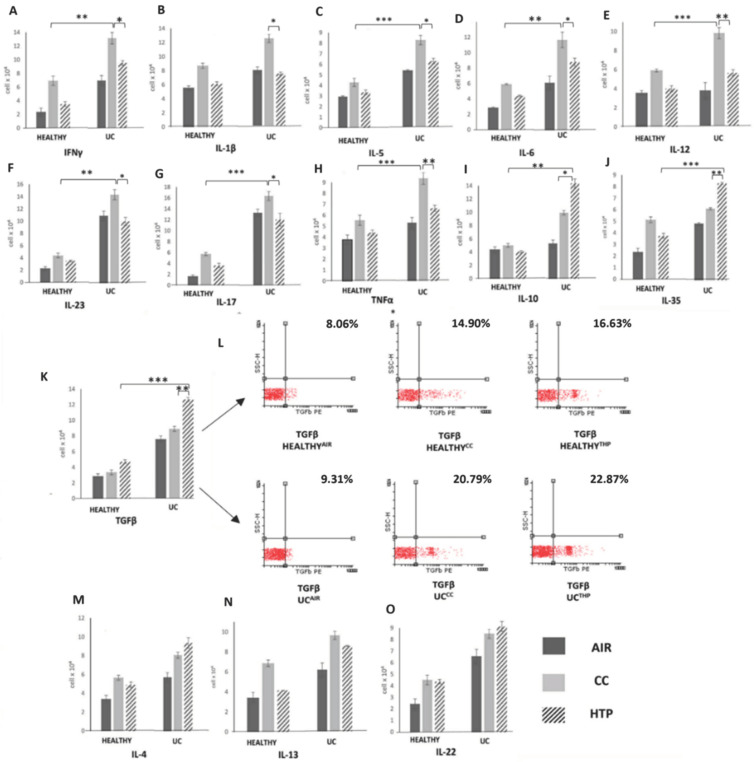
Compared to CCs, HTPs more efficiently induce the production of immunosuppressive cytokines in the immune cells of UC patients. Total numbers of IFN-γ- (**A**), IL-1β- (**B**), IL-5- (**C**), IL-6- (**D**), IL-12- (**E**), IL-23- (**F**), IL-17- (**G**), TNF-α- (**H**), IL-10- (**I**), IL-35- (**J**), TGF-β- (**K**), IL-4- (**M**), IL-13- (**N**), and IL-22-producing immune cells (**O**) in the blood of healthy volunteers and UC^CC^, UC^HTP^, and UC^AIR^ patients, including representative dot plots (**L**), demonstrate the increased presence of leukocytes producing immunosuppressive cytokines in the blood of UC^HTP^ compared to UC^CC^ patients. Values are presented as mean ± SEM; * *p* < 0.05, ** *p* < 0.01, *** *p* < 0.001.

**Figure 6:**
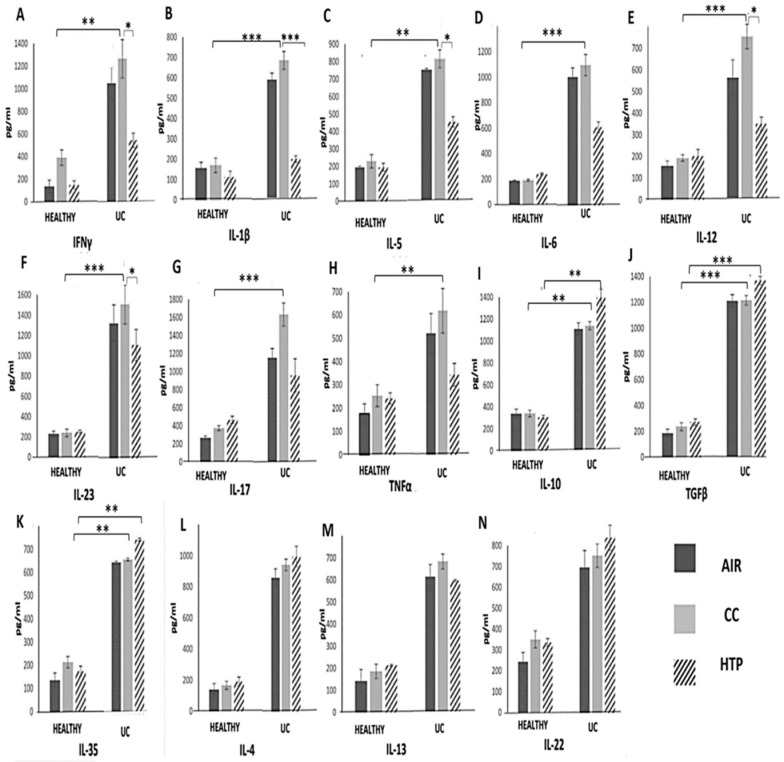
CC- and HTP-dependent effects on serum levels of inflammatory, pro-fibrotic, and immunosuppressive cytokines in healthy volunteers and UC patients. Concentrations of IFN-γ (**A**), IL-1β (**B**), IL-5 (**C**), IL-6 (**D**), IL-12 (**E**), IL-23 (**F**), IL-17 (**G**), TNF-α (**H**), IL-10 (**I**), IL-35 (**J**), TGF-β (**K**), IL-4 (**L**), IL-13 (**M**), and IL-22 (**N**) in serum samples of healthy volunteers and UC^CC^, UC^HTP^ and UC^AIR^ patients show that CCs more efficiently promoted the production of inflammatory cytokines and HTPs more efficiently induced the production of immunosuppressive and pro-fibrotic cytokines in UC patients. Values are presented as mean ± SEM; * *p* < 0.05, ** *p* < 0.01, *** *p* < 0.001.

Through the production of inflammatory and immunosuppressive cytokines, immune cells orchestrate systemic immune response, affecting UC development and progression [21,25]. Significantly increased numbers of inflammatory immune cells (Figure 5) and remarkably elevated concentrations of inflammatory cytokines (Figure 6) were observed in UC patients compared to the healthy controls. The analysis of UC patients’ blood and serum samples showed that the continuous use of either CCs or HTPs modulated the secretory properties of UC patients’ leukocytes, affecting UC progression. Significantly lower numbers of immune cells, which produce inflammatory cytokines that induce the systemic inflammatory response (IFN-γ, IL-1β, IL-5, IL-6, IL-12, IL-23, IL-17), and significantly lower concentrations of these cytokines were observed in the serum samples of UC^CC^ and UC^HTP^ compared to UC^AIR^ patients. 

Interestingly, CCs and HTPs exhibited variances in their capacity to stimulate the production of inflammatory and immunosuppressive cytokines in UC patients. Lower numbers of IFN-γ-, IL-1β-, IL-5-, IL-6-, IL-12-, IL-23-, IL-17-, and TNF-α-producing immune cells and higher numbers of IL-10-, TGF-β- and IL-35-producing immune cells were noticed in the blood of UC^HTP^ compared to UC^CC^ patients (Figure 5). Accordingly, lower levels of IFN-γ, IL-1β, IL-5, IL-6, IL-12, IL-23, IL-17, and TNF-α and higher levels of IL-10, IL-35, and TGF-β were observed in the serum samples of UC^HTP^ compared to UC^CC^ patients (Figure 6). There were no significant differences in the concentrations of IL-4, IL-13, or IL-22 between the blood and serums samples of UC^AIR^, UC^CC^, and UC^HTPs^ patients.

## 4. Discussion

Although COPD, UC, and DM develop due to the immune-cell-driven injury of parenchymal cells in the lungs, gut, or pancreatic tissue, the systemic inflammatory response is considered crucially responsible for the aggravation and progression of these diseases [1,22,23,24,25,26]. Systemic inflammation can lead to increased airway inflammation and narrowing, importantly contributing to the development of acute exacerbations that can be life-threatening for patients with severe COPD [26]. In UC patients, the systemic inflammatory response can compromise the integrity of the intestinal barrier, allowing harmful substances to enter the bloodstream. This can trigger a detrimental immune response, which aggravates the inflammatory process in the colon, increasing the risk of bowel perforation [25]. In DM patients, systemic inflammation impairs insulin sensitivity, leading to poorly controlled blood sugar levels, significantly increasing the risk of stroke, diabetic nephropathy, and retinopathy [23]. In line with these findings, identifying and addressing factors that induce systemic inflammatory response in COPD, DM, and UC patients is crucially important for disease management and for improving outcomes for individuals living with these chronic conditions [1].

Herein, we have demonstrated that the continuous use of either CCs or HTPs modulates cytokine production in circulating leukocytes, affecting systemic inflammatory response in COPD, DM, and UC patients. Long-term exposure to CC-derived smoke and HTP-sourced aerosols altered immune-cell-driven inflammation, importantly contributing to the progression of these chronic inflammatory diseases.

It is well known that CC-derived smoke contains thousands of toxic chemicals that generate ROS and cause oxidative stress in alveolar epithelial cells, inducing the enhanced release of damage-associated molecular patterns (DAMPs) and alarmins into the extracellular environment [27]. These inflammatory mediators are captured by dendritic cells (DCs), initiating detrimental immune response in the lungs [28,29]. Upon binding to Toll-like receptors (TLRs) on lung DCs, DAMPs and alarmins initiate the MyD88-driven intracellular signaling pathway and induce an enhanced activation of the transcription factors NF-κβ, IRFs, and AP1, which regulate the expression of genes that elicit the production of inflammatory cytokines (TNF-α, IL-1β, IL-6, IL-17, and IFN-γ) [23,24]. Accordingly, we observed significantly higher numbers of TNF-α-, IL-1β-, IL-6-, IL-17- and IFN-γ-producing immune cells and elevated levels of these inflammatory cytokines in the blood of COPD^CC^ compared to COPD^AIR^ patients (Figure 1 and Figure 2). Since TNF-α, IL-1β, and IL-6 are key mediators of systemic inflammation [1], we assume that the CC-dependent increased production of these cytokines was mainly responsible for the generation of detrimental immune response in COPD^CC^ patients. TNF-α, IL-1β, and IL-6 increased the expression of adhesion molecules on the lung ECs of CC users, facilitating the recruitment of immune cells into the inflamed lungs [30]. Additionally, TNF-α and IL-1β trigger an NF-kB-driven intracellular signaling cascade in lung-infiltrated Th17 lymphocytes, enhancing the production of IL-17, which promotes the production of ROS, elastases, and matrix metalloproteinases (MMPs) in neutrophils, exacerbating tissue destruction and remodeling in the lungs of COPD patients [31]. In addition to increased serum levels of TNF-α, IL-1β, and IL-6, prolonged exposure to CC-derived smoke also increased the production of IL-13, which stimulated the goblet cells to produce and secrete mucus [32]. Excessive mucus production can aggravate airway obstruction, impair mucociliary clearance, and increase susceptibility to respiratory infections in COPD^CC^ patients [33]. Additionally, the increased concentration of IL-13 could enhance collagen deposition, smooth muscle hypertrophy, and fibrosis, aggravating airway hyper-responsiveness in COPD^CC^ patients [32,33]. In line with these findings, it can be concluded that the long-term use of CCs significantly increases the risk of exacerbating and aggravating COPD. 

Compared to CCs, HTPs had an impaired capacity to induce the secretion of inflammatory, pro-Th1, and pro-Th17 cytokines, but managed to more efficiently enhance the production of immunosuppressive cytokines in COPD patients (Figure 1 and Figure 2). Although these findings may indicate that the long-term use of HTPs could be less harmful to COPD patients than CCs, it has to be highlighted that continuous exposure to HTP-derived aerosols also significantly increased the presence of TGF-β-secreting immune cells in the blood of COPD^HTP^ patients (Figure 1 and Figure 2). TGF-β plays a central role in the development of pulmonary fibrosis in response to lung injury and inflammation [34,35]. It induces epithelial-to-mesenchymal transition and stimulates the differentiation of fibroblasts into myofibroblasts, enabling the massive production and deposition of collagen in inflamed lungs [34,35]. Additionally, the excessive secretion of TGF-β may result in the formation of disorganized blood vessels in inflamed lungs, further contributing to the aggravation of lung inflammation and fibrosis [34]. Since continuous exposure to HTP-derived aerosols increased the production of pro-fibrotic TGF-β in COPD^HTP^ patients (Figure 1 and Figure 2), up-coming experimental and clinical studies should focus on investigating the effects of HTP-derived aerosols on the TGF-β-dependent development and progression of lung fibrosis.

In a similar manner as was observed in COPD patients, CCs and HTPs differed in their capacity to regulate the secretion of inflammatory and immunosuppressive cytokines in the immune cells of DM patients (Figure 3 and Figure 4). Higher numbers of leukocytes producing pro-Th1 (TNF-α, IL-12, IFN-γ) and pro-Th17 cytokines (IL-17, IL-1β, IL-6, IL-22, IL-23) were observed in the blood of DM^CC^ compared to DM^HTP^ patients (Figure 3). The NF-kB-dependent signaling pathway is activated in immune cells that are continuously exposed to CC-derived smoke [36]. By triggering an NF-kB-driven intracellular cascade in neutrophils, DCs, macrophages, and T lymphocytes, CCs enhance the secretion of inflammatory cytokines, crucially contributing to the development of systemic, Th1-, and Th17-cell-driven immune response, which aggravates on-going inflammation in the pancreatic islets [23,24,36]. In line with these findings, we noticed a positive correlation between parameters of DM progression (glycemia, HbA1c) and concentrations of Th1- and Th17-related cytokines in the serum samples of DM patients who continuously used CCs. 

The total numbers of inflammatory immune cells and the serum levels of inflammatory cytokines were also higher in DM^HTPs^ compared to DM^AIR^ patients (Figure 3 and Figure 4), suggesting that continuous exposure to HTP-derived aerosols also generated a detrimental systemic inflammatory response in DM patients. Importantly, compared to CCs, HTPs more efficiently promoted the production of immunosuppressive cytokines (IL-10 and IL-35) in the leukocytes of DM patients, and therefore, HTP-dependent systemic inflammation was weaker than the systemic immune response elicited by CC-derived smoke. In a similar manner as was observed in COPD^HTP^ patients, long-term exposure to HTP-derived aerosols enhanced the synthesis of pro-fibrotic TGF-β in the immune cells of DM^HTP^ patients, increasing the risk of the development of excessive fibrosis [34]. Additionally, the increased production of TGF-β has been linked with an increased activity of osteoclasts and with the aggravation of osteoporosis in DM patients [37]. Since DM patients have lower bone mineral density and are at a higher risk of developing osteoporosis [38], the long-term consequences of the HTP-dependent activation of the TGF-β pathway in immune cells on the development and progression of osteoporosis in DM^HTP^ patients should be explored in detail in future experimental and clinical studies.

In contrast to the findings observed in COPD and DM patients, the use of CCs and HTPs did not significantly aggravate the systemic inflammatory response in UC patients (Figure 5 and Figure 6). Continuous exposure to CC-derived smoke and HTP-derived aerosols did not increase the production of inflammatory cytokines that play a pathogenic role in the development and progression of colon injury (Figure 6). Importantly, HTPs managed to induce the expansion of IL-10- and IL-35-producing immunosuppressive immune cells in the blood of UC patients (Figure 5). Since nicotine may regulate the phenotype and function of immune cells in the gut [39], we assume that nicotine was mainly responsible for the expansion of immunosuppressive cells. By interacting with α7 nAChR on DCs and macrophages, nicotine enhances the production of anti-inflammatory IL-10, leading to the generation of immunosuppressive Tregs [40]. Furthermore, nicotine activates the α7 nAChR/PI3K/Akt/Erk1/Erk2 signaling pathway, promoting the self-renewal and proliferation of Tregs and enhancing their ability to produce immunosuppressive IL-35 [40]. 

Considering that nicotine concentration is approximately the same in HTP-derived aerosols and in CC-sourced smoke [18], we hypothesize that lower numbers of immunosuppressive immune cells in UC^CC^ compared to UC^HTP^ patients developed as a consequence of different CC- and HTP-dependent tobacco processing. The HTP-specific heating process occurs at lower temperatures than CC-associated combustion, resulting in the production of aerosols which have different compounds than CC-derived smoke [18]. CC-derived smoke contains carbon monoxide (CO) and tar [41]. CO- and tar-containing oxidants generate ROS, which induce oxidative stress, DNA damage, and apoptosis in rapidly proliferating immunosuppressive immune cells [42]. HTPs do not generate tar, and the level of CO in HTP-derived aerosols was found to be 1% of that contained in CC-sourced smoke [18]. Additionally, CC-derived smoke contains benzene, 1, 3 butadiene, acrylonitrile, and ethylene oxide, which negatively affect the proliferation of activated Tregs in the colons of UC patients [43,44]. Consequently, it is highly probable that the reduced number of immunosuppressive leukocytes in the blood of UC^CC^ compared to UC^HTP^ patients was primarily due to the toxic effects CC-derived smoke on their viability and proliferation.

In summary, due to different methods of tobacco processing, CC-sourced smoke and HTP-derived aerosols contain different chemical constituents and, therefore, possess different immunomodulatory characteristics. The toxic chemicals contained in CC-derived smoke (CO, benzene, 1, 3 butadiene, acrylonitrile, ethylene oxide, etc.) induce oxidative stress in immune cells, elicit an MAPK-signaling cascade, and activate transcriptional factors NF-kB and AP-1, which results in the enhanced production of inflammatory cytokines and in the generation of a potent systemic inflammatory response [11,12,41]. HTP-dependent tobacco processing does not involve combustion, and therefore the concentrations of all these combustion-associated toxic compounds are present at a value of less than 1% in HTP-derived aerosols [16,17]. Compared to CCs, HTPs have weaker capacity to induce the production of inflammatory cytokines, but they are able to more efficiently induce the activation of the PI3K/Erk-driven signaling pathway in circulating immune cells, resulting in the increased synthesis of immunosuppressive cytokines and pro-fibrotic TGF-β [16,17]. 

Accordingly, the long-term use of either CCs or HTPs significantly modulated cytokine production in circulating leukocytes, affecting the systemic inflammatory response in patients suffering from chronic inflammatory diseases. The continuous use of CCs and HTPs aggravated immune-cell-driven systemic inflammation in COPD and DM patients, but not in UC patients, suggesting that the immunomodulatory effects of CC-derived smoke and HTP-sourced aerosols are disease-specific. Therefore, the immunomodulatory effects of CCs and HTPs cannot be generalized, and have to be determined for particular immune-cell-driven inflammatory diseases. In this study, all members of the experimental and control groups were matched according to the frequency and duration of CC/HTP usage, as well as demographic and clinical parameters, which resulted in a relatively small number of participants in some groups, potentially affecting the statistical power of the study. Therefore, the results presented in this study should be considered as a starting point for the design and implementation of up-coming experimental and larger clinical studies, which should analyze CC- and HTP-dependent alterations in cytokine-related intracellular signaling pathways in immune cells that infiltrate particular inflamed organs in detail. 

It should be also noted that, in this study, short- and mid-term effects of CCs and HTPs on immune-cell-driven systemic inflammation were determined and that samples were obtained only from Serbian patients, suggesting limited generalizability of the findings to other populations or regions. Therefore, future clinical trials should include patients from different parts of the world and should determine the long-term consequences of CC- and HTP-dependent immunomodulation on the progression of chronic inflammatory disease.

## Data Availability

The data that support the findings of this study are available from the corresponding author upon reasonable request.

## References

[1] Chen L., Deng H., Cui H., Fang J., Zuo Z., Deng J., Li Y., Wang X., Zhao L. (2017). Inflammatory responses and inflammation-associated diseases in organs. Oncotarget.

[2] Antar S.A., Ashour N.A., Marawan M.E., Al-Karmalawy A.A. (2023). Fibrosis: Types, Effects, Markers, Mechanisms for Disease Progression, and Its Relation with Oxidative Stress, Immunity, and Inflammation. Int. J. Mol. Sci..

[3] Palomo M., Moreno-Castaño A.B., Salas M.Q., Escribano-Serrat S., Rovira M., Guillen-Olmos E., Fernandez S., Ventosa-Capell H., Youssef L., Crispi F. (2023). Endothelial activation and damage as a common pathological substrate in different pathologies and cell therapy complications. Front. Med..

[4] Xiang Y., Zhang M., Jiang D., Su Q., Shi J. (2023). The role of inflammation in autoimmune disease: A therapeutic target. Front. Immunol..

[5] Kastratovic N., Markovic V., Harrell C.R., Arsenijevic A., Stojanovic M.D., Djonov V., Volarevic V. (2023). Effects of combustible cigarettes and electronic nicotine delivery systems on the development and progression of chronic lung inflammation in mice. Nicotine Tob. Res..

[6] Pavlovic D., Miloradovic D., Stojanovic M.D., Harrell C.R., Polosa R., Rust S., Volti G.L., Caruso M., Jakovljevic V., Djonov V. (2023). Cigarette smoke attenuates mesenchymal stem cell-based suppression of immune cell-driven acute liver failure. Toxicol. Lett..

[7] Caruso M., Emma R., Distefano A., Rust S., Poulas K., Giordano A., Volarevic V., Mesiakaris K., Boffo S., Arsenijevic A. (2023). Comparative assessment of electronic nicotine delivery systems aerosol and cigarette smoke on endothelial cell migration: The Replica Project. Drug Test Anal..

[8] Russell A.E., Liao Z., Tkach M., Tarwater P.M., Ostrowski M., Théry C., Witwer K.W. (2022). Cigarette smoke-induced extracellular vesicles from dendritic cells alter T-cell activation and HIV replication. Toxicol. Lett..

[9] Lugg S.T., Scott A., Parekh D., Naidu B., Thickett D.R. (2022). Cigarette smoke exposure and alveolar macrophages: Mechanisms for lung disease. Thorax.

[10] Chen J., Wang X., Schmalen A., Haines S., Wolff M., Ma H., Zhang H., Stoleriu M.G., Nowak J., Nakayama M. (2023). Antiviral CD8+ T-cell immune responses are impaired by cigarette smoke and in COPD. Eur. Respir. J..

[11] Qiu F., Liang C.L., Liu H., Zeng Y.Q., Hou S., Huang S., Lai X., Dai Z. (2017). Impacts of cigarette smoking on immune responsiveness: Up and down or upside down?. Oncotarget.

[12] White P.C., Hirschfeld J., Milward M.R., Cooper P.R., Wright H.J., Matthews J.B., Chapple I.L.C. (2018). Cigarette smoke modifies neutrophil chemotaxis, neutrophil extracellular trap formation and inflammatory response-related gene expression. J. Periodontal Res..

[13] Shaykhiev R., Krause A., Salit J., Strulovici-Barel Y., Harvey B.G., O’Connor T.P., Crystal R.G. (2009). Smoking-dependent reprogramming of alveolar macrophage polarization: Implication for pathogenesis of chronic obstructive pulmonary disease. J. Immunol..

[14] Thatcher T.H., Benson R.P., Phipps R.P., Sime P.J. (2008). High-dose but not low-dose mainstream cigarette smoke suppresses allergic airway inflammation by inhibiting T cell function. Am. J. Physiol.-Lung Cell Mol. Physiol..

[15] Ortiz-Quintero B., Martínez-Espinosa I., Pérez-Padilla R. (2022). Mechanisms of Lung Damage and Development of COPD Due to Household Biomass-Smoke Exposure: Inflammation, Oxidative Stress, MicroRNAs, and Gene Polymorphisms. Cells.

[16] Simonavicius E., McNeill A., Shahab L., Brose L.S. (2019). Heat-not-burn tobacco products: A systematic literature review. Tob. Control.

[17] Znyk M., Jurewicz J., Kaleta D. (2021). Exposure to Heated Tobacco Products and Adverse Health Effects, a Systematic Review. Int. J. Environ. Res. Public Health.

[18] St Helen G., Jacob Iii P., Nardone N., Benowitz N.L. (2018). IQOS: Examination of Philip Morris International’s claim of reduced exposure. Tob. Control.

[19] Kastratovic N., Cekerevac I., Sekerus V., Markovic V., Arsenijevic A., Volarevic A., Harrell C.R., Jakovljevic V., Djonov V., Volarevic V. (2024). Effects of combustible cigarettes and heated tobacco products on immune cell-driven inflammation in chronic obstructive respiratory diseases. Toxicol. Sci..

[20] Acovic A., Simovic Markovic B., Gazdic M., Arsenijevic A., Jovicic N., Gajovic N., Jovanovic M., Zdravkovic N., Kanjevac T., Harrell C.R. (2018). Indoleamine 2,3-dioxygenase-dependent expansion of T-regulatory cells maintains mucosal healing in ulcerative colitis. Therap. Adv. Gastroenterol..

[21] Volarevic V., Zdravkovic N., Harrell C.R., Arsenijevic N., Fellabaum C., Djonov V., Lukic M.L., Simovic Markovic B. (2019). Galectin-3 Regulates Indoleamine-2,3-dioxygenase-Dependent Cross-Talk between Colon-Infiltrating Dendritic Cells and T Regulatory Cells and May Represent a Valuable Biomarker for Monitoring the Progression of Ulcerative Colitis. Cells.

[22] Rajabi H., Mortazavi D., Konyalilar N., Aksoy G.T., Erkan S., Korkunc S.K., Kayalar O., Bayram H., Rahbarghazi R. (2022). Forthcoming complications in recovered COVID-19 patients with COPD and asthma; possible therapeutic opportunities. Cell Commun. Signal.

[23] Rohm T.V., Meier D.T., Olefsky J.M., Donath M.Y. (2022). Inflammation in obesity, diabetes, and related disorders. Immunity.

[24] Solt L.A., Burris T.P. (2015). Th17 cells in Type 1 diabetes: A future perspective. Diabetes Manag..

[25] Tatiya-Aphiradee N., Chatuphonprasert W., Jarukamjorn K. (2018). Immune response and inflammatory pathway of ulcerative colitis. J. Basic Clin. Physiol. Pharmacol..

[26] Groenewegen K.H., Postma D.S., Hop W.C., Wielders P.L., Schlösser N.J., Wouters E.F., COSMIC Study Group (2008). Increased systemic inflammation is a risk factor for COPD exacerbations. Chest.

[27] Cha S.R., Jang J., Park S.M., Ryu S.M., Cho S.J., Yang S.R. (2023). Cigarette Smoke-Induced Respiratory Response: Insights into Cellular Processes and Biomarkers. Antioxidants.

[28] Cook P.C., MacDonald A.S. (2016). Dendritic cells in lung immunopathology. Semin. Immunopathol..

[29] Piccinini A.M., Midwood K.S. (2010). Dampening inflammation by modulating TLR signalling. Mediat. Inflamm..

[30] Shyam Prasad Shetty B., Chaya S.K., Kumar V.S., Mahendra M., Jayaraj B.S., Lokesh K.S., Ganguly K., Mahesh P.A. (2021). Inflammatory Biomarkers Interleukin 1 Beta (IL-1β) and Tumour Necrosis Factor Alpha (TNF-α) Are Differentially Elevated in Tobacco Smoke Associated COPD and Biomass Smoke Associated COPD. Toxics.

[31] Kubysheva N., Boldina M., Eliseeva T., Soodaeva S., Klimanov I., Khaletskaya A., Bayrasheva V., Solovyev V., Villa-Vargas L.A., Ramírez-Salinas M.A. (2020). Relationship of Serum Levels of IL-17, IL-18, TNF-α, and Lung Function Parameters in Patients with COPD, Asthma-COPD Overlap, and Bronchial Asthma. Mediat. Inflamm..

[32] Cooper P.R., Poll C.T., Barnes P.J., Sturton R.G. (2010). Involvement of IL-13 in tobacco smoke-induced changes in the structure and function of rat intrapulmonary airways. Am. J. Respir. Cell Mol. Biol..

[33] Choudhury P., Biswas S., Singh G., Pal A., Ghosh N., Ojha A.K., Das S., Dutta G., Chaudhury K. (2022). Immunological profiling and development of a sensing device for detection of IL-13 in COPD and asthma. Bioelectrochemistry.

[34] Saito A., Horie M., Nagase T. (2018). TGF-β Signaling in Lung Health and Disease. Int. J. Mol. Sci..

[35] Fernandez I.E., Eickelberg O. (2012). The impact of TGF-β on lung fibrosis: From targeting to biomarkers. Proc. Am. Thorac. Soc..

[36] Chung S., Sundar I.K., Hwang J.W., Yull F.E., Blackwell T.S., Kinnula V.L., Bulger M., Yao H., Rahman I. (2011). NF-κB inducing kinase, NIK mediates cigarette smoke/TNFα-induced histone acetylation and inflammation through differential activation of IKKs. PLoS ONE.

[37] Kum J.J.Y., Howlett C.J., Khan Z.A. (2022). Dysregulated transforming growth factor-beta mediates early bone marrow dysfunction in diabetes. Commun. Biol..

[38] Wongdee K., Charoenphandhu N. (2011). Osteoporosis in diabetes mellitus: Possible cellular and molecular mechanisms. World J. Diabetes.

[39] Mahmoudzadeh L., Abtahi Froushani S.M., Ajami M., Mahmoudzadeh M. (2023). Effect of Nicotine on Immune System Function. Adv. Pharm. Bull..

[40] Nakata Y., Miura K., Yamasaki N., Ogata S., Miura S., Hosomi N., Kaminuma O. (2022). Expression and Function of Nicotinic Acetylcholine Receptors in Induced Regulatory T Cells. Int. J. Mol. Sci..

[41] Talhout R., Schulz T., Florek E., van Benthem J., Wester P., Opperhuizen A. (2011). Hazardous compounds in tobacco smoke. Int. J. Environ. Res. Public Health.

[42] Chen X., Song M., Zhang B., Zhang Y. (2016). Reactive Oxygen Species Regulate T Cell Immune Response in the Tumor Microenvironment. Oxidative Med. Cell. Longev..

[43] Soeteman-Hernández L.G., Bos P.M., Talhout R. (2013). Tobacco smoke-related health effects induced by 1,3-butadiene and strategies for risk reduction. Toxicol. Sci..

[44] Berkowitz L., Schultz B.M., Salazar G.A., Pardo-Roa C., Sebastián V.P., Álvarez-Lobos M.M., Bueno S.M. (2018). Impact of Cigarette Smoking on the Gastrointestinal Tract Inflammation: Opposing Effects in Crohn’s Disease and Ulcerative Colitis. Front. Immunol..

